# Prognostic significance of neutrophil-to-lymphocyte ratio in patients with malignant pleural mesothelioma: a meta-analysis

**DOI:** 10.18632/oncotarget.15404

**Published:** 2017-02-16

**Authors:** Nan Chen, Shuai Liu, Lin Huang, Wanling Li, Wenhao Yang, Tianxin Cong, Lin Ding, Meng Qiu

**Affiliations:** ^1^ West China School of Medicine, West China Hospital, Sichuan University, Chengdu, China; ^2^ Department of Medical Oncology, Cancer Center, The State Key Laboratory of Biotherapy, West China Hospital, Sichuan University, Guoxue Alley, Chengdu, China

**Keywords:** inflammation, malignant pleural mesothelioma, neutrophil-to-lymphocyte ratio, prognosis, meta-analysis

## Abstract

Systemic inflammation responses can be reflected by peripheral blood count and combine index like the neutrophil-to-lymphocyte (NLR). The NLR has been reported to be a poor prognostic indicator in cancer recently. However, the prognostic effect of the NLR in patients with malignant pleural mesothelioma (MPM) still unclear yet. We conducted this meta-analysis aiming to evaluate the pooled value of NLR in prognosis as well as clinical characteristics in malignant pleural mesothelioma. A total of 11 studies with 1533 patients were included in this meta-analysis, in which 10 studies investigated the prognosis role of NLR using hazard ratio (HR) and 95% confidence intervals (95% CI). The elevated NLR was detected to be associated with a poor overall survival (OS)(HR=1.48, 95%CI=1.16-1.89, *P* < 0.001). The significant prognostic roles of NLR were also indicated in subgroup analyses. NLR level was also associated with histology instead of gender, stage or performance status (PS) score. These findings suggested that the elevated NLR could be a potential prognostic factor for malignant pleural mesothelioma patients and might be associated with histology as an efficient clinical index to stratify patients.

## INTRODUCTION

Malignant pleural mesothelioma (MPM) is a high aggressive tumor originates from mesothelial surfaces with a poor prognosis and a median survival of 9 to 13 months [1], and there were still no reliable prognostic indicators. Several prognostic biomarkers have been reported in the literature which including hemoglobin and white blood cell counts, performance status according to the Eastern Cooperative Oncology Group (ECOG) scale, age, weight loss, disease stage and imaging results (tumor volume, metabolic activity) to distinguish patients with good prognosis from those with inferior outcomes [2, 3]. Although these results for the prognosis helpful, but they were far from enough to predict the survival outcomes. Therefore, studies to further identify novel prognostic factors associated with accuracy and efficiency calls for the exertion of clinicians.

Inflammatatory responses have been found associated with tumor progression, evidence also shown that increased systemic inflammation was associated with poor overall survival (OS) in numerous types of cancer [4–9]. Inflammation is a crucial component of tumor microenvironment [8]. Inflammatory cells in the tumor microenvironment have important effects on tumor development, and markers of systemic inflammation may provide significant information for prognostication. Accumulating evidence suggested inflammation-related cells correlated closely with cancer are including neutrophils [10, 11], platelets [12], and lymphocytes [13]. Neutrophil to lymphocyte ratio (NLR), calculated as a simple ratio between neutrophil and lymphocyte counts, an index of systemic inflammation, has been related to poor survival for a variety of malignant tumors as renal cell carcinoma, colorectal cancer, hepatocellular carcinoma, prostate cancer and gastric cancer [14–18].

Therefore, it is a reasonable assumption that these two factors together might be applied as a prognostic factor. Here, we conducted a meta-analysis aiming to further verify the pooled prognostic value of NLR in malignant pleural mesothelioma.

## RESULTS

### Study characteristics and qualities

**Figure 1:**
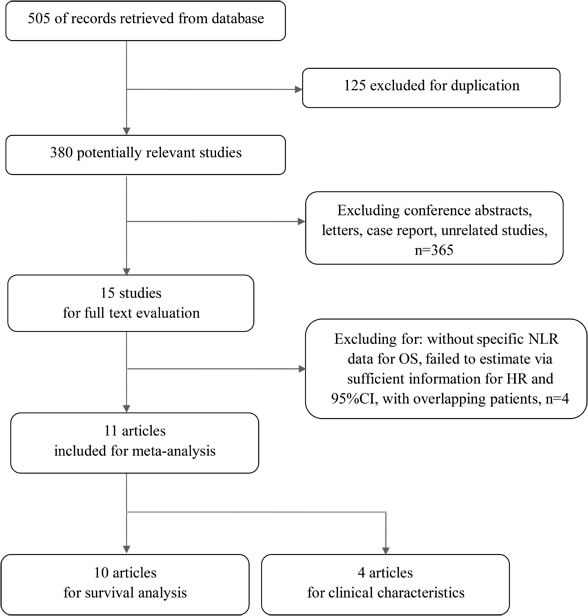
The flow chart of study selection NLR: neutrophil-to-lymphocyte; OS: overall survival; HR: hazard ratio; CI: confidence intervals.

The flow chart of selection process was shown in Figure 1. We initially retrieved 380 studies in total without duplicates. After further screening, 365 studies were excluded for conference abstracts, case reports, reviews, or studies unrelated to this meta-analysis. Then, 15 studies were identified for the next step of evaluation with full text. During the further analysis, 4 studies [19, 20, 21, 22] were ruled out because they were failed to present specific NLR data for OS, not available in neither univariate nor multivariate analysis, or failed to estimate via sufficient information for HR and 95%CI, or studies with overlapping patients. Thus, finally 11 studies with 1533 patients were included in our meta-analysis, in which 10 studies [23–32] valuated the association between NLR and survival of malignant pleural mesothelioma patients, four study [23, 27, 29, 31] were included for investigation of NLR and patients’ clinical characteristics. The publication time ranged from 2010 to 2016. The ethnicities of the studies contained Asian and Caucasian. We extracted HR and 95%CI from multivariate analysis in seven studies and univariate analysis in three studies. All included studies reported data for OS. The study quality was evaluated via Newcastle-Ottawa scale, and the scores of the 11 studies ranged from 6 to 8. The main characteristics were shown in Table 1.

**Table 1:** The baseline characteristics of included studies

Author	Year	Country	Ethnicity	*N*^a^	Study design	Stage	Treatment	Cut-off value	Analysis	NOS score
Abakay	2014	Turkey	Caucasian	155	Retrospective	NA	NA	3	NA	8
Cedres	2014	Spain	Caucasian	52	Retrospective	III/IV	Chemotherapy/best supportive care	5	MV	6
Cihan	2014	Turkey	Caucasian	50	Retrospective	I/II/III/IV	Surgery/Chemotherapy/Radiotherapy	3	UV	6
Hooper	2015	the UK	Caucasian	73	Prospective	I/II/III/IV	Chemotherapy/best supportive care	4	MV	8
Kao	2013	Australia	Caucasian	148	Retrospective	I/II/III/IV	Surgery/Chemotherapy/Radiotherapy	3	MV	6
Kao	2011	Australia	Caucasian	85	Retrospective	I/II/III/IV	Surgery/neoadjuvant chemotherapy	3	MV	7
Kao	2010	Australia	Caucasian	173	Retrospective	I/II/III/IV	First-line/second/third-line chemotherapy	5	MV	8
Meniawy	2013	Australia	Caucasian	274	Retrospective	I/II/III/IV	Surgery/Chemotherapy/best supportive care	5	MV	7
Pinato	2012	the UK	Caucasian	171	Retrospective	I/II/III/IV	Chemotherapy/best supportive care	5	MV	8
Tanrikulu	2015	Turkey	Caucasian	202	Retrospective	I/II/III/IV	Chemotherapy/best supportive care	3	MV	6
Yamagishi	2015	Japan	Asian	150	Retrospective	I/II/III/IV	Surgery/Chemotherapy/Radiotherapy/best supportive care	5	UV	6

^a^ Number of included patients

NA: not available; OS: overall survival; MV: multivariate analysis; UV: univariate analysis; NOS: Newcastle-Ottawa Quality Assessment Scale.

### NLR and survival

**Figure 2:**
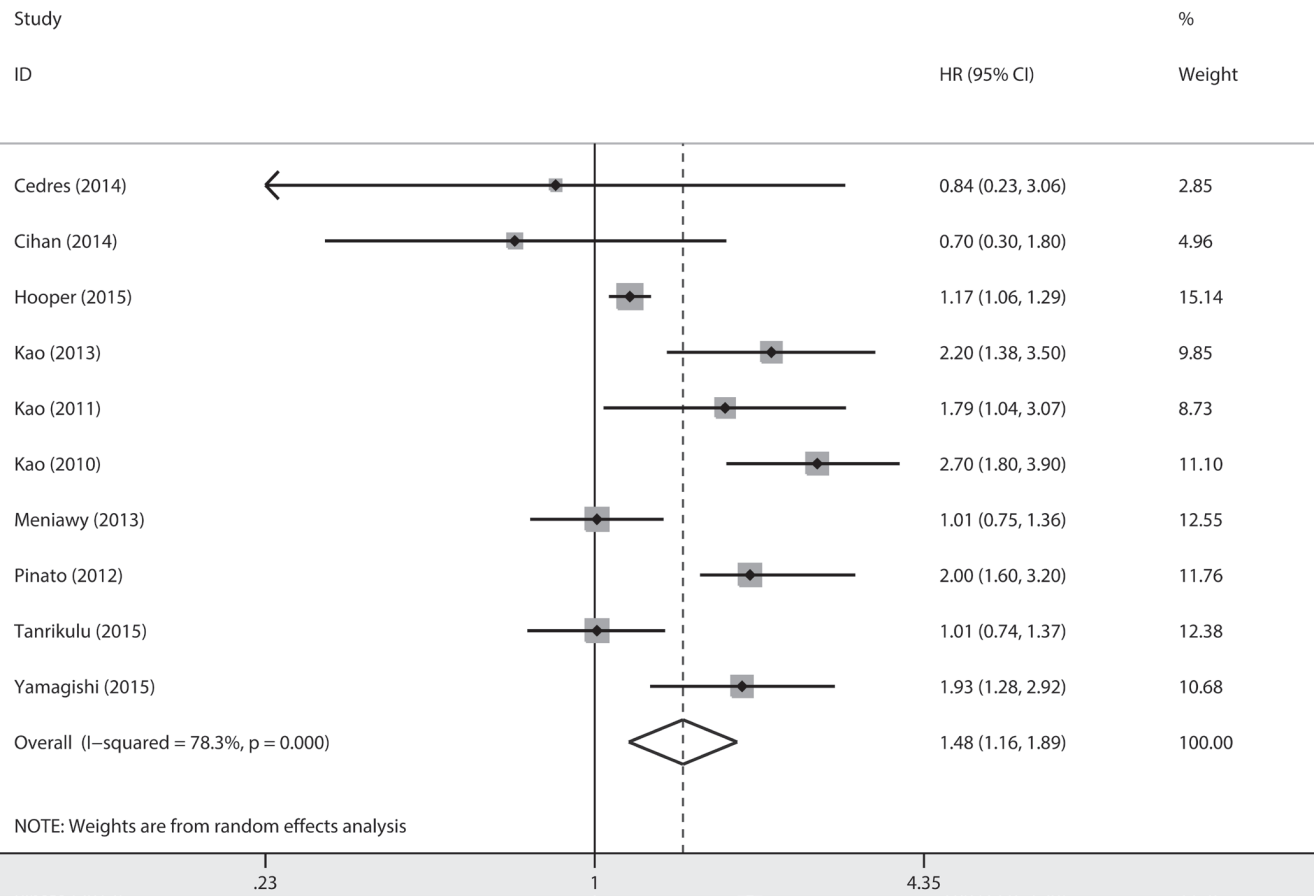
Forest plot of the association between NLR and OS in MPM overallly The elevated NLR was detected to be significantly associated with a poor OS. HR: hazard ratio; CI: confidence intervals.

There were 7 studies with multivariate analysis and 3 studies with univariate analysis providing information regarding OS. Overall, an elevated NLR was significantly associated with a poor OS (HR=1.48, 95%CI=1.16-1.89, P=0.001)(Figure 2). The significance was detected in studies with multivariate analysis (HR=1.50, 95%CI=1.15-1.95, P=0.003). We further detected a significant association between NLR and OS in studies with Caucasian population (HR=1.44, 95%CI=1.11-1.86, P=0.006), larger sample size (HR=1.58, 95%CI=1.07-2.35, P=0.022), higher study quality (HR = 1.579, 95%CI=1.12-2.22, P=0.009) and cut-off value of five (HR=1.68, 95%CI=1.10-2.56, P=0.015). The detailed results were shown in Table 2 and the extracted data were presented in Supplementary table S1.

**Table 2:** The pooled data on survival of meta-analysis

Variables	*N*^a^	Case^b^	Pooled data		Heterogeneity	
			HR(95%CI)	*P*	*I*^2^	*Ph*
**Overall**	10	1378	1.483(1.164-1.889)	0.001	78.3%	<0.001
**Ethnicity**						
Caucasian	9	1228	1.437(1.112-1.857)	0.006	78.6%	<0.001
Asian	1	150	1.930(1.278-2.915)	0.002	NA	NA
**Study design**						
Retrospective	9	1305	1.537(1.138-2.076)	0.005	75.5%	<0.001
Prospective	1	73	1.170(1.065-1.286)	0.001	NA	NA
**Survival analysis**						
Multivariate	8	1178	1.499(1.151-1.953)	0.003	80.4%	<0.001
Univariate	2	200	1.261(0.473-3.362)	0.644	75.4%	0.044
**Sample size**						
≤149	5	408	1.368(0.948-1.973)	0.094	62.2%	0.032
>149	5	970	1.583(1.067-2.349)	0.022	84.6%	<0.001
**Cut-off value**						
3	4	485	1.358(0.844-2.185)	0.207	71.9%	0.014
4	1	73	1.170(1.065-1.286)	0.001	NA	NA
5	5	820	1.681(1.104-2.560)	0.015	79.5%	0.001
**NOS score**						
≤6	5	602	1.343(0.866-2.082)	0.188	69.5%	0.011
>6	5	776	1.579(1.123-2.221)	0.009	85.6%	<0.001

^a^ Numbers of studies included in the meta-analysis

^b^ Number of patients of included studies

NA: not available; OS: overall survival; HR: hazard ratio; 95%CI: confidence interval; P: *p* value of pooled HR; *I*^2^: value of Higgins I-squared statistics; *Ph*: *p* value of Heterogeneity test.

### NLR and clinical characteristics

As for clinical characteristics, we detected NLR level was associated with histology (odds ratio (OR)=0.59, 95%CI=0.40-0.86, P=0.005), patients with non-epithelioid histological subtype are more likely in an elevated NLR. However, we failed to observe the association between NLR level with gender, tumor stage and performance status (PS) score. These results were presented in Table 3 and detailed extracted data were shown in Supplementary table S2.

**Table 3:** The pooled data on clinical characteristics of included studies

Variables	*N*^a^	Case^b^	Pooled data		Heterogeneity	
			OR(95%CI)	*P*	*I*^2^	*Ph*
Gender	3	488				
Male			Reference			
Female			0.793(0.516-1.219)	0.290	0.0%	0.369
Histology	4	526				
Epithelial			Reference			
Non-epithelial			0.588(0.404-0.855)	0.005	37.3%	0.188
Stage	2	205				
I/II			Reference			
III/IV			0.677(0.342-1.339)	0.262	0.0%	0.521
PS score	2	302				
0			Reference			
≧1			0.652(0.379-1.120)	0.121	0.0%	0.410

^a^ Numbers of studies included in the meta-analysis

^b^ Number of patients of included studies

PS score: performance status score; OR: odds ratio; 95%CI: confidence interval; P: *p* value of pooled HR; *I*2: value of *X*2 based I-squared statistics; *Ph*: p value of Heterogeneity test.

### Sensitivity analysis and publication bias

**Figure 3:**
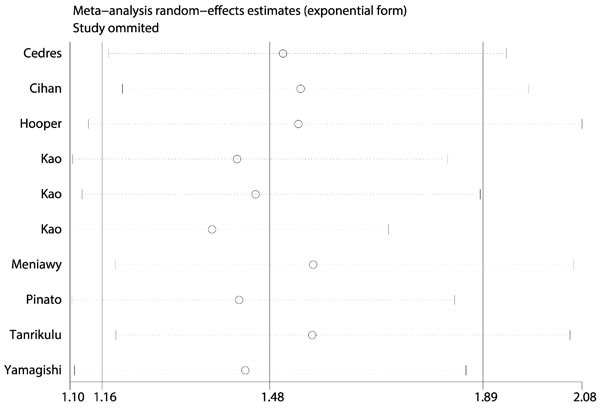
Sensitivity analysis of included studies to evaluate the stability of our results We did not observe any variations of the results, which proved the stability of results of our meta-analysis.

**Figure 4:**
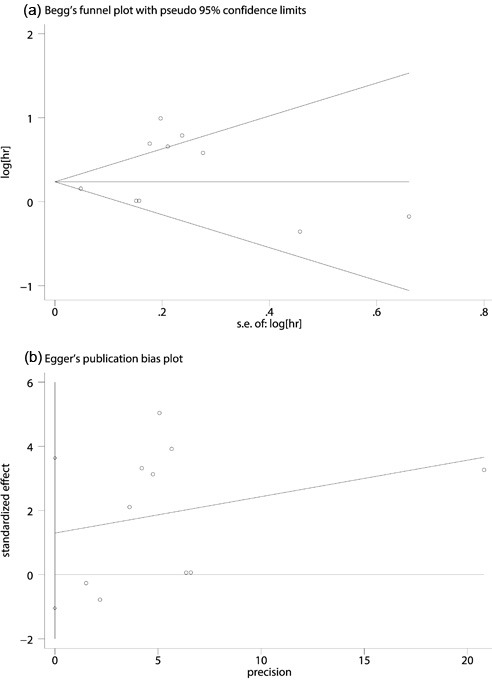
Begg's funnel plot (a) and Egger's linear regression tests (b) for the assessment of potential publication bias We did not detected any evidence of publication bias for OS.

Sensitivity analysis was performed and we did not observe any variations of the results, which proved the stability of results of our meta-analysis (Figure 3). In addition, The Begg's funnel plot (Pr>|z|=0.474) (Figure 4a) and the Egger's test (P>|t|=0.237) (Figure 4b) did not detected any evidence of publication bias for OS, as well as for clinical characteristics.

## DISCUSSION

Systemic inflammatory response is important in cancer progresssion [33]. Inflammation-relatated cells involved in the constitution and regulation of tumer cell microenvironment. It may act as an intermediary between tumor cell and inflammatory. Different peripheral blood count may reflect the body's inflammatory response, and NLR as an combined index may reflect the balance between neutrophils and immunocytes, making it a prognostic factor. Several studies have demonstrated that elevated NLR is related with a poor prognosis in MPM, but the result still controversial. Thus we conducted this meta-analysis and it was the first to indicate that a high NLR was associated with poor survival of patients with MPM.

Similar to our study, a recent clinical research published in 2016 investigated the relation between NLR and malignant pleural mesothelioma [22]. In their research they collected and analysised 36 patients’ clinical information, and didn't detect any significant correlation bettwen NLR and MPM prognosis. Thus we could find that NLR on prognosis of patients with malignant pleural mesothelioma was still a question worth exploring. These studies just as we mentioned above, might not be able to draw reliable conclusions with the limitation of sample size. Our research using the method of meta-analysis contained a larger sample size, and might be more credible and provide more reliable information compared to other single clinical research.

Base on the result of our meta-analysis, we found that an elevated NLR was significantly associated with a poor OS. Furthermore, results were proved in subgroup analysis stratified by ethnicity, study design, suvival analysis, cut-off value and NOS score. In addition, relationship between NLR and clinical characters including histology, gender, tomur stageand performance status (PS) score were also accessed. NLR level was associated with histology of malignant pleural mesothelioma that it was higher in patients with a nonepithelioid histological subtype. However, we failed to detected any association between NLR level with gender, tumor stage and performance status (PS) score.

Our study were in correspondence with previous studies which investigated the prognosis value of NLR in other kind of tumors such as liver cancer [34], colorectal cancer [35], pancreatic cancer [36], etc. These study also demonstrated that an elevated NLR was important in predicting prognosis before treatment. Thus it was believed to be a promising marker playing role in cancer diagnosis, prognosis prediction and individual treatment in future. However, the optimal cut-off value for NLR in predicting the prognosis of gastric cancer remains unclear [37]. The cut-off values in our analysis ranged from 3 to 5, and they were determined by the median value of all patients, or on the basis of previous studies. To establish a suitable cut-off value, we performed subgroup analyses with the cut-off values. Most included studies applied five as cut-off value and significant survival outcomes were detected in subgroup stratified patients by five.

Our results were not consistent with some published studies, we didn't find the association between NLR level and tumor stage. A possible explanation for this might be that the degree of inflamation is different from patients in different tumor stage. Several studies had shown that malignant pleural tumors were always secondary to asbestosis exposion, and early pleural tumors were more easily associated with acute infection and inflammation effusion. By contrast, the chance of acute inflammatory response in advanced pleural tumor patients is less. Unfortunately, patients with the presence of active infection were more likely to be excluded from the study in patients selection process, which may lead to a negative result. More studies were warranted on this filed in future.

Our study has some limitations. First, although we made comprehensive search, there were only 11 studies included and the limited patients numbers may have influence on the outcomes, thus futher studies at a large scale might be needed to confirm the prognosis value of NLR in MPM. In addiation, heterogeneity was a potential problem that may reduce the accurary of the results. Difference in baseline features of included studies such as histology, treatment, cut-off value of NLR and follow-up period, etc might potentially have influence in pooled results. Finally, some original articles evaluated the prognostic effect of NLR in univariate analysis, whereas others had data of multivariate analysis, which may contribute to some bias of the pooled data.

In conclusion, our study demonstrated that the elevated NLR predicted poor survival of MPM. And NLR also might be associated with histology of MPM. The NLR could be useful for predicting survival of patients with MPM. Further studies were warranted to conform the exact value of NLR in the prognosis of MPM.

## MATERIALS AND METHODS

### Search strategy

A systemic search was conducted in PubMed, Embase and web of Science databases update to Jun 26, 2016. The following words were applied as search terms: “neutrophil-to-lymphocyte”, “neutrophil-lymphocyte ratio”, “neutrophil/lymphocyte ratio”, “NLR”, “malignant pleural mesothelioma”, “malignant mesothelioma”, “pleural mesothelioma”, “MPM”, “MM”. There was no language restriction of our literature search. The references of identified articles were retrieved manually for potential eligible studies.

### Inclusion and exclusion criteria

Studies met the following criteria were considered eligible: (1) the studies investigated the association between NLR and prognosis or clinical characteristics of MPM patients; (2) for prognosis, hazard ratio (HR) and 95% CI were reported, or Kaplan-Meier curve and relevant information were available to estimate HR and 95% CI. (3) for clinical characteristics, odds ratio (OR) and 95% CI were used to measure the association. We excluded studies if they: (1) were conference abstracts, letters, case reports; (2) failed to report failed to present specific NLR data for OS in neither univariate nor multivariate analysis, or failed to estimate via sufficient information for HR and 95%CI, or NLR as a continue variable; (3) presented to be duplicate publications. When studies with overlapping patients were met, only the study with the most patients was included. Two investigators (Nan Chen and Shuai Liu) reviewed the identified studies independently and a final discussion was launched to reach a consistency.

### Data extraction and quality assessment

Two investigators(Nan Chen and Lin Huang) extracted information independently using a standard data extraction table. The following information were recorded for each included study: name of first author, year of publication, ethnicity, number of recruited patients, treatment of patient, follow-up time, analysis method, type of survival, cut-off value for NLR, clinical characteristics information, HR and 95% CI for survival. The Newcastle-Ottawa Quality Assessment Scale (NOS) was used to assess the quality of each included study independently by these two investigators. NOS score more than 6 were considered as high-quality studies. The two investigators discussed to reach a consensus when there was any disagreement. The Newcastle-Ottawa Quality Assessment Scale (NOS) we used was shown in Supplementary table S3 and the detailed scores of each included study was presented in Supplementary table S4.

### Statistics analysis

In this meta-analysis, OR with 95%CI was used to evaluate the association between NLR and clinical characteristics. As for prognosis, HR and 95%CI were extracted from each study to calculate the pooled HR (high level vs. low level). When the study reported both univariate and multivariate results, we chose multivariate analysis for final calculation. While the HR and 95%CI were not reported directly, Kaplan-Meier curve and relevant data were collected to estimate related HR with 95%CI. Test of heterogeneity was conducted by Cochran's Q test and Higgins I-squared statistic. P>0.10 and I2<50% were regarded as no significant heterogeneity and the fixed-effects model was used. Otherwise, the random-effects model was applied. In addition, further subgroup analyses to explore potential heterogeneity were performed stratified by ethnicity, sample size, study design, survival analysis, NOS score and cut-off value. We further conducted sensitivity to evaluate the stability of the results. Additionally, Begg's funnel plot and Egger's linear regression tests were performed to access publication bias. All the analyses were carried out by STATA 12.0 (STATA Corporation, College Station, TX, USA).

## SUPPLEMENTARY TABLES


