# Supplementary material for: Prognostic significance of neutrophil-to-lymphocyte ratio in patients with malignant pleural mesothelioma: a meta-analysis
Source: Oncotarget. 2017 Feb 16;8(34):57460–9. doi: 10.18632/oncotarget.15404 (PMC5593657; doi:10.18632/oncotarget.15404)
Supplement: Supplementary file 1 [file oncotarget-08-57460-s001.doc]

**Prognostic Significance of Neutrophil-to-Lymphocyte Ratio in Patients with Malignant Pleural Mesothelioma: A Meta-Analysis**

Nan Chen^1, a^, Shuai Liu^1, a^, Lin Huang^1, a^, Wanling Li^1^, Wenhao Yang^1^, Tianxin Cong^1^, Lin Ding^1^, Meng Qiu^1,2,*^

^1^ West China School of Medicine/West China Hospital, Sichuan University, Chengdu, 610041, China

^2^ Department of Medical Oncology, Cancer Center, the State Key Laboratory of Biotherapy, West China Hospital, Sichuan University, No. 37, Guoxue Alley, Chengdu, Sichuan, 610041, China

^*^ Corresponding author: Meng Qiu

Address: No. 37, Guoxue Alley, Chengdu, Sichuan, 610041, China

E-mail: [qiumeng33@hotmail.com](mailto:qiumeng33@hotmailmail.com)

^a^ These authors contributed equally to this work

**Supplementary table S1** The extracted data on survival of included studies

| **First Author** | **Year** | **N^a^** | **Cut-off value** | **HR** | **95%CI** | |
| --- | --- | --- | --- | --- | --- | --- |
|  |  |  |  |  | **ll** | **ul** |
| **OS** |  |  |  |  |  |  |
| Abakay | 2014 | 155 | 3 | 1.67 | 1.14 | 2.46 |
| Cedres | 2014 | 52 | 5 | 0.84 | 0.23 | 3.06 |
| Cihan | 2014 | 50 | 3 | 0.70 | 0.30 | 1.80 |
| Hooper | 2015 | 73 | 4 | 1.17 | 1.08 | 1.29 |
| Kao | 2013 | 148 | 3 | 2.20 | 1.38 | 3.50 |
| Kao | 2011 | 85 | 3 | 1.79 | 1.04 | 3.07 |
| Kao | 2010 | 173 | 5 | 2.70 | 1.80 | 3.90 |
| Meniawy | 2013 | 274 | 5 | 1.01 | 0.75 | 1.36 |
| Pinato | 2012 | 171 | 5 | 2.00 | 1.60 | 3.20 |
| Tanrikulu | 2015 | 202 | 3 | 1.01 | 0.74 | 1.37 |
| Yamagishi | 2015 | 150 | 5 | 1.93 | 1.28 | 2.92 |

^a^ Number of included patients

OS: overall survival; HR: hazard ratio; 95%CI: confidence interval; ll: lower limit; ul: upper limit.

**Supplementary table S2** The extracted data on clinical characteristics of included studies

| **Study** | **Year** | **N^a^** | |
| --- | --- | --- | --- |
|  |  | **Low level^b^** | **High level^b^** |
| **Gender** |  |  |  |
| **Male** |  |  |  |
| Abakay | 2014 | 37 | 24 |
| Kao | 2010 | 76 | 61 |
| Pinato | 2012 | 94 | 49 |
| **Female** |  |  |  |
| Abakay | 2014 | 45 | 43 |
| Kao | 2010 | 19 | 12 |
| Pinato | 2012 | 15 | 13 |
| **Histology** |  |  |  |
| **Epithelioid** |  |  |  |
| Abakay | 2014 | 61 | 36 |
| Kao | 2013 | 18 | 28 |
| Kao | 2010 | 65 | 41 |
| Pinato | 2012 | 73 | 38 |
| **Non-epithelioid** |  |  |  |
| Abakay | 2014 | 21 | 31 |
| Kao | 2013 | 8 | 13 |
| Kao | 2010 | 20 | 29 |
| Pinato | 2012 | 29 | 1 |
| **Stage** |  |  |  |
| **I / II** |  |  |  |
| Kao | 2010 | 6 | 3 |
| Pinato | 2012 | 34 | 14 |
| **III / IV** |  |  |  |
| Kao | 2010 | 32 | 36 |
| Pinato | 2012 | 52 | 28 |
| **PS score** |  |  |  |
| **0** |  |  |  |
| Kao | 2010 | 21 | 14 |
| Pinato | 2012 | 36 | 12 |
| **≧1** |  |  |  |
| Kao | 2010 | 70 | 57 |
| Pinato | 2012 | 56 | 36 |

^a^ Number of patients

^b^ Number of patients with low NLR level or high NLR level

PS score: performance status score;

**Supplementary table S3** Newcastle - Ottawa quality assessment scale for cohort studies

NEWCASTLE - OTTAWA QUALITY ASSESSMENT SCALE

COHORT STUDIES

Note: A study can be awarded a maximum of one star for each numbered item within the Selection and Outcome categories. A maximum of two stars can be given for Comparability

**Selection (Max 4 Stars)**

1) Representativeness of the exposed cohort

a) truly representative of the average _______________ (describe) in the community *****

b) somewhat representative of the average ______________ in the community *

c) selected group of users eg nurses, volunteers

d) no description of the derivation of the cohort

2) Selection of the non exposed cohort

a) drawn from the same community as the exposed cohort *

b) drawn from a different source *

c) no description of the derivation of the non exposed cohort

3) Ascertainment of exposure

a) secure record (eg surgical records) *

b) structured interview *

c) written self report

d) no description

4) Demonstration that outcome of interest was not present at start of study

a) yes *

b) no

**Comparability** **(Max 2 Stars)**

1) Comparability of cohorts on the basis of the design or analysis

a) study controls for _____________ (select the most important factor) *

b) study controls for any additional factor * (This criteria could be modified to indicate specific control for a second important factor.)

**Outcome (Max 3 Stars)**

1) Assessment of outcome

a) independent blind assessment *

b) record linkage *

c) self report

d) no description

2) Was follow-up long enough for outcomes to occur

a) yes (select an adequate follow up period for outcome of interest) *

b) no

3) Adequacy of follow up of cohorts

a) complete follow up - all subjects accounted for *

b) subjects lost to follow up unlikely to introduce bias - small number lost > 20 % (select an adequate follow up, or description provided of those lost) *

c) follow up rate < 80% and no description of those lost

d) no statement

**Supplementary table S4** The detailed NOS scores of included studies

| Study ID | First author | Study design | Publication year | NEWCASTLE - OTTAWA QUALITY ASSESSMENT SCALE | | | | | | | | |
| --- | --- | --- | --- | --- | --- | --- | --- | --- | --- | --- | --- | --- |
|  |  |  |  | Selection | | | | Comparabilitiy | Outcome | | | Total |
|  |  |  |  | Q1 | Q2 | Q3 | Q4 | Q1 | Q1 | Q2 | Q3 |  |
| 1 | Abakay | retrospestive | 2014 | 1 | 1 | 1 | 1 | 2 | 1 | 1 | 0 | 8 |
| 2 | Cedres | retrospestive | 2014 | 1 | 1 | 1 | 1 | 0 | 1 | 1 | 0 | 6 |
| 3 | Cihan | retrospestive | 2014 | 1 | 1 | 1 | 1 | 0 | 1 | 1 | 0 | 6 |
| 4 | Hooper | prospestive | 2015 | 1 | 1 | 1 | 1 | 1 | 1 | 1 | 1 | 8 |
| 5 | Kao | retrospestive | 2013 | 0 | 1 | 1 | 1 | 1 | 1 | 0 | 1 | 6 |
| 6 | Kao | retrospestive | 2011 | 0 | 1 | 1 | 1 | 1 | 1 | 1 | 1 | 7 |
| 7 | Kao | retrospestive | 2010 | 1 | 1 | 1 | 1 | 2 | 1 | 0 | 1 | 8 |
| 8 | Meniawy | retrospestive | 2013 | 1 | 1 | 1 | 1 | 1 | 1 | 1 | 0 | 7 |
| 9 | Pinato | retrospestive | 2012 | 1 | 1 | 1 | 1 | 2 | 1 | 0 | 1 | 8 |
| 10 | Tanrikulu | retrospestive | 2015 | 1 | 1 | 1 | 1 | 0 | 1 | 0 | 1 | 6 |
| 11 | Yamagishi | retrospestive | 2015 | 1 | 1 | 1 | 1 | 0 | 1 | 0 | 1 | 6 |
